# Establish VO_2_max prediction models based on exercise and body parameters from the step test

**DOI:** 10.7150/ijms.109977

**Published:** 2025-05-28

**Authors:** Chia-An Ho, Hung-Chih Yeh, Hei-Tung Lau, En-Yu Chang, Chih-Wen Hsu, Chun-Hao Chang, Chi-Chang Huang, Wen-Sheng Chang Chien, Chin-Shan Ho

**Affiliations:** Graduate Institute of Sports Science, National Taiwan Sport University, Taoyuan, Taiwan

**Keywords:** cardiorespiratory fitness assessment, maximal oxygen uptake prediction model, step test

## Abstract

This study addresses the challenge of cardiorespiratory fitness (CRF) assessment by proposing predictive models for maximal oxygen uptake (VO₂max) based on step test parameters. Recognizing VO₂max as a gold standard for CRF evaluation, this study aims to develop a VO₂max prediction model based on a step test, providing a simple and practical alternative for primary healthcare and health monitoring. This model enables clinicians and health management professionals to efficiently assess patients' cardiorespiratory fitness. Through the recruitment of 200 healthy Taiwanese adults, the research combined direct VO₂max measurements with step test heart rate (HR) data and variables like age, sex, percentage body fat (PBF), body mass index (BMI), and resting heart rate (RHR) to develop six predictive models. This method is applicable for clinical health monitoring, cardiorespiratory fitness assessment in patients with chronic diseases, and exercise capacity monitoring in cardiac rehabilitation programs. The study identified that PBF-based models consistently outperformed BMI-based ones, with Model^PBF3^, which incorporates HR responses during exercise, achieving the highest accuracy (R² = 0.689; SEE = 4.6971 ml·kg⁻¹·min⁻¹). These results indicate that the model can effectively estimate VO₂max and be applied in primary healthcare, remote health monitoring, and cardiac rehabilitation settings, providing a simple and practical tool for cardiorespiratory fitness assessment in clinical practice. Validation via PRESS cross-validation and Bland-Altman plots confirmed the stability and reliability of the models across diverse subgroups. By bridging the gap between laboratory-grade precision and everyday practicality, the study introduces a robust, low-cost, and user-friendly tool for CRF assessment, adaptable for non-athletes and those unable to perform high-intensity exercises. This research advances the feasibility of CRF self-management in varied settings, while future iterations could extend its applicability to broader demographics and integrate additional physiological variables for universal adoption.

## Introduction

Cardiorespiratory fitness (CRF) is closely associated with cardiovascular health, metabolic syndrome, and all-cause mortality, making it a crucial indicator for health assessment 1,2,3. Among CRF indicators, maximal oxygen uptake (VO₂max) is considered the gold standard, as it reflects an individual's oxygen consumption capacity at maximal exercise intensity 4. However, direct VO₂max measurement requires high-intensity exercise and specialized equipment, making it costly and less accessible for routine assessments 5,6. To overcome these limitations, researchers have developed various submaximal exercise tests, such as the 6-minute walk test, 12-minute run test, and step test 7,8,9. These methods have lower equipment requirements and are suitable for individuals across different age groups and health conditions, leading to their widespread use in CRF assessment 10,11,12. Since the mid-20th century, numerous step test protocols have been developed to accommodate diverse populations. Among them, the YMCA 3-minute step test and Harvard step test have been extensively applied in fitness evaluations and cardiac rehabilitation 7,8,9,10. These tests incorporate factors such as heart rate (HR), age, sex, and body composition to establish VO₂max prediction models 11,12.

The step test has been developed since the mid-20th century, with various protocols designed to accommodate different step rates, heights, and durations, catering to diverse age groups, health conditions, and fitness levels. Classic tests such as the “Young Men's Christian Association (YMCA) Three-Minute Step Test” and the “Harvard Step Test” have been widely utilized for fitness assessment and cardiac rehabilitation evaluation 7,8,9,10. The outcomes of these tests are closely associated with factors such as heart rate, age, sex, and body composition. Studies have demonstrated that post-exercise heart rate recovery effectively evaluates individual CRF and serves as a basis for predicting VO₂max through heart rate-based models 11,12. As understanding of various physiological indicators has advanced, researchers have incorporated additional variables, such as percentage body fat (PBF) and body mass index (BMI), into prediction models to improve accuracy 13,14,15. Typically, individuals with better CRF exhibit faster post-exercise HR recovery 16,17,18. Research indicates a significant linear relationship between VO₂max and HR changes during and after exercise 19,20. Faster HR recovery in individuals with higher CRF is attributed to superior cardiovascular adaptability and enhanced muscular metabolic efficiency 21,22. Consequently, HR recovery has become a non-invasive method for evaluating CRF, particularly well-suited for simple step tests 23. Studies suggest that HR indices and the difference in HR between specific time points (e.g., the difference between the 3rd minute of exercise and 1 minute post-exercise) can serve as effective responses in prediction models. These responses enhance the reliability of step test predictions under various scenarios 24,25.

Despite the use of various HR response indicators for CRF evaluation, there remains room for improvement in the accuracy and generalizability of step test models. Previous studies have identified age, sex, BMI, and PBF as critical factors influencing VO₂max 26,27. Consequently, recent research has increasingly incorporated additional variables to enhance model precision. Studies have shown that integrating multiple HR parameters can not only improve the explanatory power of CRF variability but also reduce the standard error of prediction in models 28,29,30. To address this, the current study employs a multiple linear regression analysis approach, combining physiological variables and exercise HR parameters to develop a VO₂max prediction model for the step test. Cross-validation methods are widely utilized in model validation to assess stability. In this study, the effectiveness of the model was evaluated using predicted residual error sum of squares (PRESS) cross-validation, comparing its applicability and stability across groups of different sex, age, and CRF levels 16,31,32. Additionally, Bland-Altman analysis was employed to assess the agreement between measured and estimated values, ensuring the reliability of the predictions 33.

In modern society, influenced by environmental factors, the demand for family health management is steadily increasing, and simple yet accurate tools for CRF self-assessment have become a pressing need 26,34. The step test, characterized by its simplicity, low cost, and high safety, is suitable for people of various age groups and health conditions. Designing a VO₂max prediction model for the step test that is applicable in-home settings not only facilitates autonomous health management but also provides a more practical assessment tool for clinical use. Currently, few studies differentiate VO₂max prediction formulas based on economic feasibility, convenience, and accuracy. Such distinctions are crucial to enabling individuals to select prediction methods that best suit their conditions. Against this backdrop, this study develops VO₂max prediction formulas based on physical characteristics, RHR, and exercise test parameters. The study also validates and compares the effectiveness of different models in predicting VO₂max. We hypothesize that exercise-induced HR recovery during the step test is a potential predictor of VO₂max. By incorporating HR parameters alongside age, sex, and physical characteristics (PBF/BMI) into the VO₂max prediction formula, we aim to enhance the accuracy of VO₂max prediction.

## Methods

### Study design

In this study, all participants were required to complete two exercise tests: direct VO₂max measurement and the step test. VO₂max for both the training and testing groups was measured using an electromagnetically braked bicycle ergometer (Excalibur Sport Ergometer, Lode BV, the Netherlands) in conjunction with a Cardiopulmonary Exercise Testing System (Vmax Encore 29 System, VIASYS Healthcare Inc., Yorba Linda, CA). A Polar H10 Heart Rate Monitor (Polar Electro Oy, Finland) was used to measure participants' exercise HR during the step test. Since heart rate (HR) changes during exercise are significantly correlated with measured VO₂max 35,36, this study incorporates HR Recovery (post-exercise heart rate recovery rate) as one of the VO_2_max prediction factors. HR Recovery can be further divided into (1) parasympathetic reactivation during the first 2 minutes and (2) metabolic recovery during the last 2 minutes. Using these measurements, the study developed multiple linear regression equations for predicting VO₂max based on age, sex, body composition, and with or without HR parameters. PRESS cross-validation procedures were employed to validate the prediction models. The study procedures were approved by the Institutional Review Board of National Taiwan Sport University (Taoyuan City, Taiwan) and informed consent was provided by all participants before the experiment. This study was conducted in full conformance with the relevant guidelines and regulations, i.e., the principles of the Declaration of Helsinki guidelines. The study was registered at http://rad.ntsu.edu.tw/ (reference number: NTSUIRB-112-029). We recruited volunteers with four kinds of physical fitness characteristics using an open, independent, and random method.

### Participants

All participants (Taiwanese adults) were openly, independently, and randomly recruited through advertisements posted in public spaces. Individuals with cardiovascular, pulmonary, or metabolic diseases, or those with neurological, muscular, or skeletal conditions that could affect their ability to complete the exercise tests, were excluded. The selection criteria for participants in this study included the absence of known cardiovascular, respiratory, metabolic, neuromuscular, or musculoskeletal diseases, as well as no recent exercise-related injuries, ensuring their ability to complete the test. No restrictions were placed on participants' exercise habits or baseline fitness levels, as the primary objective was to enhance the model's applicability across individuals with varying fitness conditions. Ultimately, 200 healthy adults (age: 20-64 years; women: 50%, men: 50%, We employed a random allocation method during sample distribution to ensure representativeness and randomness in both model development and validation processes. However, stratification by sex was not applied, which may have contributed to slight gender imbalances between the training and testing groups.) completed the study. Only participants who completed the step test without interruption were included in the analysis. Ten participants were excluded based on this criterion. While minimal, this exclusion may introduce a slight selection bias and limit generalizability to populations with lower exercise tolerance. Anthropometric and body composition parameters measured for each participant included height, weight, BMI, and PBF (Table 1). Body weight and PBF were assessed using a body composition analyzer (InBody ® 770, Biospace, Inc., Seoul, Korea) 37. BMI was calculated as weight in kilograms divided by the square of height in meters.

### Maximal graded exercise test

VO₂max was measured using an electromagnetically braked bicycle ergometer in conjunction with a cardiopulmonary exercise testing system. During the test, participants wore a Polar H10 Heart Rate Monitor chest strap to monitor their HR and a Hans-Rudolph gas collection mask connected to sampling tubes and a digital flow sensor. This setup allowed for breath-by-breath measurement of air volume and analysis of O₂ and CO₂ composition. The test began with an initial workload of 25 W, increasing by 15 W every 2 minutes until participants could no longer maintain a pedaling frequency of 70 rpm. Fatigue levels during the test were assessed using the Borg Rating of Perceived Exertion (RPE, 6-20 scale). In this study, VO₂max was defined as the highest 30-second average relative oxygen uptake. Participants were considered to have achieved VO₂max if at least three of the following criteria were met: (1) VO₂ plateaued despite an increase in workload, (2) Respiratory exchange ratio (RER) ≥ 1.10, (3) Maximum HR reached at least 90% of age-predicted HRmax (i.e., 220 - age), (4) RPE ≥ 18 5.

### Step test

Before the step test, participants were equipped with a Polar H10 Heart Rate Monitor chest strap to measure HR during the exercise. The step height was set at 35 cm. During the test, participants were required to follow a stepping rhythm and ensure each step was completed in time with the cadence, stepping fully onto the platform. The step test began at a cadence of 96 SPM, with participants stepping up and down once every four beats. The stepping sequence follows "right foot up, left foot up, right foot down, left foot down", with a metronome used to maintain a consistent rhythm. During the test, participants must not lean on handrails or walls and are required to wear standard athletic attire and sports shoes. The exercise lasted for 3 minutes, and HR was measured at the end of the test. For safety reasons, if participants were unable to maintain the stepping cadence during the test, it was terminated, and their data were excluded from the analysis. HR was recorded at the start of the exercise, at the 1st, 2nd, and 3rd minutes during exercise, and at the 1st, 2nd, 3rd, and 4th minutes post-exercise. In this study, resting heart rate (RHR) refers to the heart rate measured while the participant is in a completely relaxed and stationary state, whereas exercise start heart rate (HR^0^) is recorded when the participant is standing and preparing to begin exercise. Due to postural changes and psychological factors, HR^0^ may be higher than RHR. After completing the test, all participants remained seated during heart rate recovery measurements to minimize the influence of postural changes on HR Recovery results.

### Statistical Analysis

All values were expressed as mean ± standard deviation (SD). One-way ANOVA test analysis was used to compare the difference in physical characteristics between the training and testing groups. Pearson's correlation coefficients were calculated to analyze the relationships between the independent variables of the training group (i.e., age, sex, PBF, BMI, HR parameters) and measured VO₂max, validating the effectiveness of the VO₂max prediction model. The absolute values of the correlation coefficients (r) were interpreted as follows: 0.00-0.10 as negligible correlation, 0.10-0.39 as weak correlation, 0.40-0.69 as moderate correlation, 0.70-0.89 as strong correlation, and 0.90-1.00 as very strong correlation 38. Multiple linear regression analysis with cross-validation (70% of the sample for model development and 30% for validation) was employed to construct the VO₂max prediction model. Variables included age, sex, PBF, and HR parameters. The accuracy of the VO₂max prediction equations was assessed using the coefficient of determination (R²), absolute standard error of estimate (SEE), and relative SEE (%SEE). The PRESS method was applied to validate the VO₂max prediction models 16. Bland-Altman plots were used to evaluate the agreement between predicted VO₂max values and directly measured VO₂max values 39. Statistical analyses were performed using SPSS software (version 22, IBM Corp., Armonk, NY, USA), with a significance level set at *p* < 0.05.

## Results

Table 1 presents the study population and physical characteristics of the training and testing groups, including age, height, body weight, BMI, PBF, VO₂max and resting heart rate (RHR), there is no significant difference between the training and testing groups in the total values.

Table 2 presents the Pearson correlations between measured VO₂max and the independent variables in the training group. The results indicate significant negative correlations between VO₂max and age (r = -0.409, *p* < 0.001), PBF (r = -0.738, *p* < 0.001), RHR (r = -0.241, *p* < 0.001), HR2 (r = -0.188, *p* < 0.05), HR3 (r = -0.245, *p* < 0.001), and HR4 (r = -0.175, *p* < 0.05). A significant positive correlation was observed between sex (women = 0, men = 1) and VO₂max (r = 0.603, *p* < 0.001).

Tables 3 and 4 present the multiple regression models predicting VO_2_max and the results of cross-validation. Among the PBF and BMI models, Model^PBF3^ demonstrated the highest multiple correlation coefficient and the lowest standard error of estimate (SEE) (R^2^ = 0.689, SEE = 4.6971). When predicting VO_2_max using age, sex, and body composition, the inclusion of RHR increased R^2^ from 0.654 to 0.673 and reduced SEE from 4.9190 to 4.7945 ml·kg⁻¹·min⁻¹ for Model^PBF2^, and increased R^2^ from 0.587 to 0.622 and reduced SEE from 5.3750 to 5.1554 ml·kg⁻¹·min⁻¹ for Model^BMI2^. This indicates that the explained variance of VO_2_max increased by 3.06% and 5.96% for Model^PBF2^ and Model^BMI2^, respectively, while SEE decreased by 2.53% and 4.09%, respectively. The addition of RHR and exercise HR parameters further increased the explained variance of VO_2_max by 2.38% in Model^PBF3^ and 4.66% in Model^BMI3^, with a reduction in SEE by 2.03% for Model^PBF3^ and 3.10% for Model^BMI3^. When substituting BMI with PBF for body composition parameters, R^2^ increased from 0.587 to 0.654 and SEE decreased from 5.3750 to 4.9190 ml·kg⁻¹·min⁻¹ for Model^PBF1^; R^2^ Increased from 0.622 to 0.673 and SEE decreased from 5.1554 to 4.7945 ml·kg⁻¹·min⁻¹ for Model^PBF2^; and R^2^ increased from 0.651 to 0.689 and SEE decreased from 4.9958 to 4.6971 ml·kg⁻¹·min⁻¹ for Model^PBF3^.

**Figure 1:**
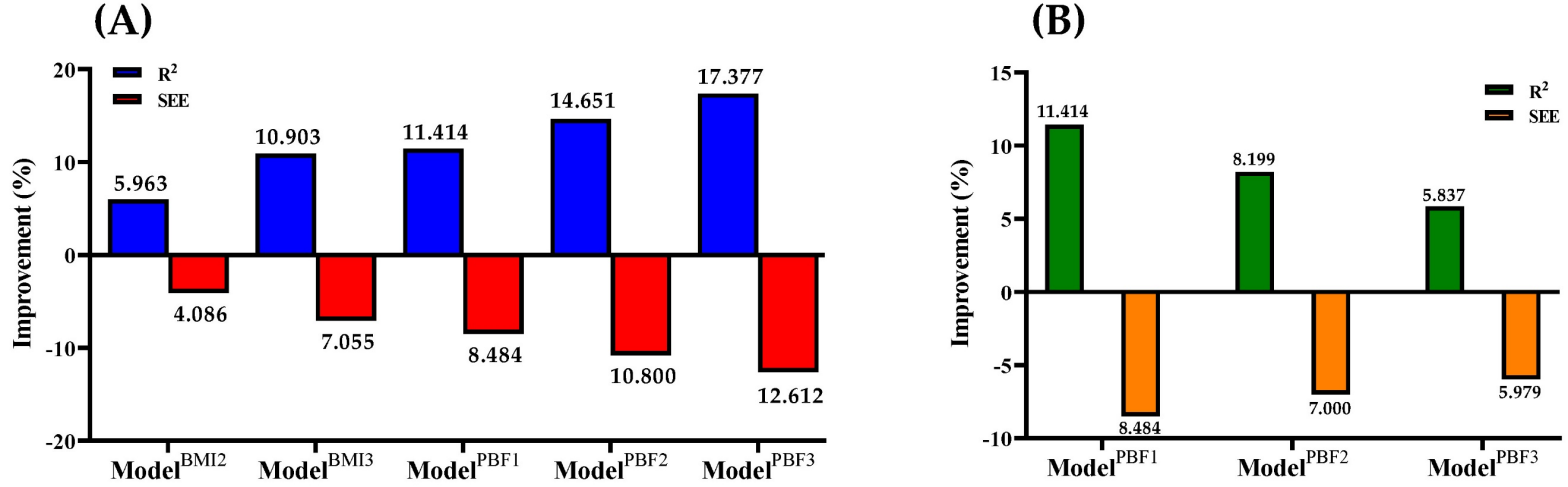
** (A)** Compared to Model^BMI1^, Model^BMI2^, Model^BMI3^, Model^PBF1^, Model^PBF2^, and Model^PBF3^ showed improved accuracy in predicting VO₂max. **(B)** The improvements in VO₂max prediction accuracy were for Model^PBF1^ compared to Model^BMI1^, Model^PBF2^ compared to Model^BMI2^, and Model^PBF3^ compared to Model^BMI3^.

These results represent increases in explained variance of VO_2_max by 11.41%, 8.20%, and 5.84%, and decreases in SEE by 8.48%, 7.00%, and 5.98% for Model^PBF1^, Model^PBF2^, and Model^PBF3^, respectively (Figure 1B). The PRESS cross-validation results indicate that changes in R^2^ and SEE for all VO_2_max prediction models were minimal (ΔR^2^ < 0.06, ΔSEE < 0.2802 ml·kg⁻¹·min⁻¹).

**Figure 2:**
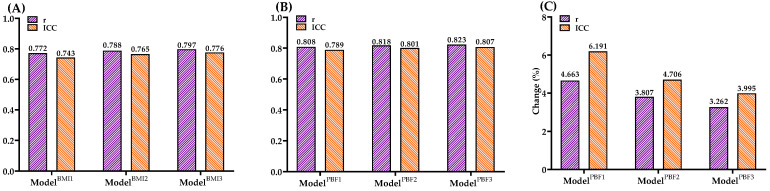
** (A)** Predictive validity (r) and reliability (ICC) of ModelBMI1, ModelBMI2, and ModelBMI3 for VO2max. **(B)** Predictive validity (r) and reliability (ICC) of ModelPBF1, ModelPBF2, and ModelPBF3 for VO2max. **(C)** Percentage changes in predictive validity (r) and reliability (ICC) comparing ModelPBF1 vs. ModelBMI1, ModelPBF2 vs. ModelBMI2, and ModelPBF3 vs. ModelBMI3 for VO2max. ICC, intraclass correlation coefficient.

Figure 2 illustrates the validity analysis (r) and reliability analysis (ICC) for the six models predicting VO₂max. As shown in Figure 2B, the models using PBF as the body composition parameter—Model^PBF1^ (r = 0.808, ICC = 0.789, both *p* < 0.001), Model^PBF2^ (r = 0.818, ICC = 0.801, both *p* < 0.001), and Model^PBF3^ (r = 0.823, ICC = 0.807, both *p* < 0.001)—demonstrated good validity and reliability in predicting VO₂max 38,40. In comparison, models using BMI as the body composition parameter—Model^BMI1^ (r = 0.772, ICC = 0.743), Model^BMI2^ (r = 0.788, ICC = 0.765), and Model^BMI3^ (r = 0.797, ICC = 0.776)—showed slightly lower validity and reliability (all *p* < 0.001; Figure 2A). The predictive validity of Model^PBF1^, Model^PBF2^, and Model^PBF3^ improved by 4.663%, 3.807%, and 3.262%, respectively, compared to their BMI-based counterparts. Similarly, the reliability of these models increased by 6.191%, 4.706%, and 3.995%, respectively (Figure 2C). These findings suggest that incorporating PBF as a body composition parameter enhances both the predictive validity and reliability of VO₂max estimation models.

**Figure 3:**
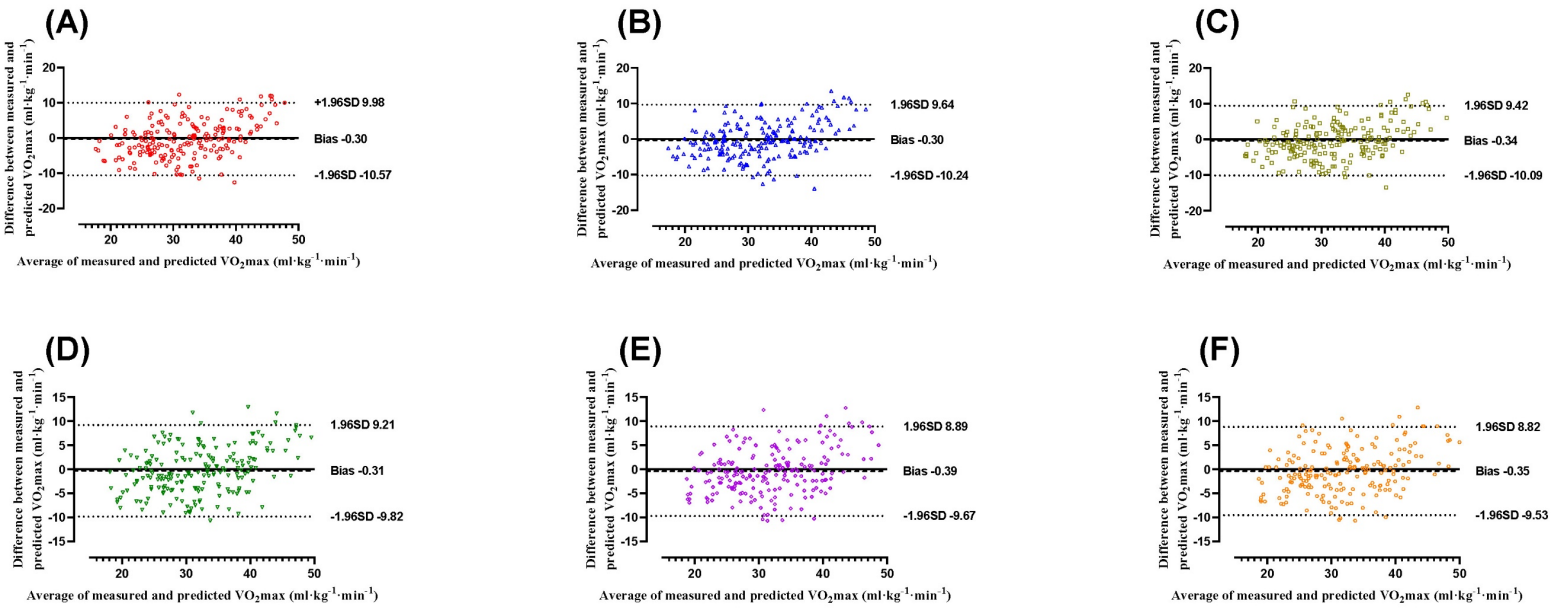
Bland-Altman Plots comparing the differences between measured and estimated VO_2_max obtained from Model^BMI1^ **(A)**, Model^BMI2^ **(B)**, Model^BMI3^ **(C)**, Model^PBF1^ **(D)**, Model^PBF2^ **(E)**, Model^PBF3^ **(F)** in the entire sample (*n* = 200). The mean differences and 95% limits of agreement are shown as dashed lines, respectively.

Figure 3 illustrates the Bland-Altman plots showing the differences between measured and predicted VO₂max values for the six models, including both BMI-based (Model^BMI1^, Model^BMI2^, and Model^BMI3^) and PBF-based models (Model^PBF1^, Model^PBF2^, and Model^PBF3^), along with their 95% limits of agreement (LoAs). For the BMI-based models, Model^BMI1^ had a mean bias of -0.30 ± 5.24 ml·kg⁻¹·min⁻¹ with LoAs ranging from -10.57 to 9.98 ml·kg⁻¹·min⁻¹ (Figure 3A), Model^BMI2^ demonstrated a mean bias of -0.30 ± 5.07 ml·kg⁻¹·min⁻¹ with LoAs between -10.24 and 9.64 ml·kg⁻¹·min⁻¹ (Figure 3B), and Model^BMI3^ showed a mean bias of -0.34 ± 4.98 ml·kg⁻¹·min⁻¹, with LoAs from -10.09 to 9.42 ml·kg⁻¹·min⁻¹ (Figure 3C). For the PBF-based models, Model^PBF1^ exhibited a mean bias of -0.31 ± 4.86 ml·kg⁻¹·min⁻¹ and LoAs between -9.82 and 9.21 ml·kg⁻¹·min⁻¹ (Figure 3D). Model^PBF2^ showed a mean bias of -0.39 ± 4.74 ml·kg⁻¹·min⁻¹, with LoAs ranging from -9.67 to 8.89 ml·kg⁻¹·min⁻¹ (Figure 3E). Similarly, Model^PBF3^ demonstrated a mean bias of -0.35 ± 4.68 ml·kg⁻¹·min⁻¹, with LoAs between -9.53 and 8.82 ml·kg⁻¹·min⁻¹ (Figure 3F). The results indicate that all models produced mean biases within the acceptable range, confirming the reliability of their predictions 41.

However, the PBF-based models consistently exhibited smaller mean biases and narrower limits of agreement compared to the BMI-based models, suggesting that incorporating PBF as a body composition parameter enhances the accuracy and consistency of VO₂max predictions.

## Discussion

Previous studies have shown that CRF is closely associated with coronary heart disease and all-cause mortality 1,42. Low CRF is linked to an increased risk of cardiovascular diseases and mortality 43. VO₂max is commonly used as a key indicator to evaluate CRF and serves as a clinically relevant tool for classification 44. Therefore, developing simple and reliable methods for home-based CRF assessments is essential. Several researchers have proposed VO₂max prediction formulas. For instance, Lee et al. developed a formula based on age, sex, height, weight, and recovery HR, achieving R² values of 0.56-0.61 and SEE values of 4.74-5.01 ml·kg⁻¹·min⁻¹ 10. Similarly, Hong et al. created two formulas using age, sex, weight, and HR recovery, explaining 73.4% and 72.2% of VO₂max variability, with SEE values of 4.7 ml·kg⁻¹·min⁻¹ 7. Compared to previous step test-based VO₂max prediction models, this study introduces several key improvements. Prior studies primarily used age, sex, and post-exercise heart rate recovery as the main variables, with a regression explanatory power ranging from 0.56 to 0.74. In contrast, our model incorporates additional heart rate variables during exercise (HR^1^, HR^3^, HR^4^) and PBF, enhancing predictive accuracy. The PBF model (Model^PBF3^) achieved an R² of 0.689 and a SEE of 4.6971 ml·kg⁻¹·min⁻¹. However, there is a lack of finer distinctions among these formulas in terms of cost, convenience, and accuracy. Such distinctions are critical for enabling individuals to choose the most suitable prediction method based on their needs. Against this backdrop, this study developed six VO₂max prediction formulas based on physical characteristics, resting HR, and exercise test parameters. These formulas were validated and compared for their predictive accuracy. As hypothesized, the results revealed significant correlations between exercise HR parameters and VO₂max (Table 2). Simple models based on age, sex, and body composition (Model^BMI1^ and Model^PBF1^) showed relatively low accuracy (R² = 0.587; R² = 0.654). Adding RHR improved the predictive accuracy (Model^BMI2^: R² = 0.622; Model^PBF2^: R² = 0.673). The highest accuracy was achieved by incorporating exercise test parameters (Model^BMI3^: R² = 0.651; Model^PBF3^: R² = 0.689). The data demonstrate that PBF is a better predictor than BMI. Compared to BMI-based models, the three PBF-based models exhibited higher coefficients of determination (R²) and lower SEE values (Figure 1B). This difference may be attributed to the fact that PBF provides a more specific measure of body fatness, whereas BMI does not differentiate between fat and lean mass, potentially leading to misclassification in individuals with atypical body composition. Among them, Model^PBF3^ was the most accurate model for predicting VO₂max in healthy adults. However, this model requires both body fat percentage and multiple heart rate measurements, which may limit its feasibility in resource-limited settings. In contrast, simpler models like Model^BMI1^ or Model^PBF1^—although slightly less accurate—are easier to implement and may be preferable for large-scale screenings or field-based applications. However, BMI-based models are more economical and practical, providing an accessible option for individuals to assess their CRF. Depending on personal circumstances, individuals can select an appropriate VO₂max prediction formula to better understand their CRF status. The results of this study indicate that the PBF prediction model (Model^PBF3^) demonstrates greater accuracy in VO₂max prediction compared to the BMI model (Model^BMI3^), aligning with previous research findings 1. However, due to the ease of obtaining BMI, it can still serve as a practical alternative indicator, particularly in large-scale health screenings and primary healthcare settings.

In standard CRF tests, the HR of non-athletes often approaches their age-predicted HRmax 45. Generally, HR reflects individual physical fitness and exercise intensity, with higher CRF levels associated with lower baseline HR values and shorter HR recovery times following cardiopulmonary exercise tests 46. Linear relationships have been observed between VO₂max and HR changes before, during, and after exercise 10,35,36,47. Thus, monitoring exercise HR can significantly enhance the predictive capacity of VO₂max models, making it a crucial factor for VO₂max estimation. Matsuo et al. reported negative correlations between HR during and after exercise and VO₂max, with the combined HR index showing a stronger correlation with VO₂max 36. Similarly, Chung et al. found a positive correlation between the difference in HR at the third minute of a 3-min step test and recovery HR with VO₂max. In line with previous findings, this study demonstrated significant negative correlations between VO₂max and HR^2^ (r = -0.188, *p* < 0.05), HR^3^ (r = -0.245, *p* < 0.001), and HR^4^ (r = -0.175, *p* < 0.05) during the step test (Table 2) 35. These results suggest that HR recovery during exercise testing can be considered a key factor for assessing CRF in adults. By monitoring HR responses during the step test, it is possible to objectively evaluate participants' physiological load during exercise and further develop VO₂max prediction formulas.

Previous studies have demonstrated that age, sex, and body characteristics (BMI or PBF) are significant predictors of VO₂max 10,36, which aligns with the findings of this study. In the simplest VO₂max prediction formulas developed here, based on age, sex, and BMI/PBF, the explained variance of VO₂max was 58.7% for Model^BMI1^ and 65.4% for Model^PBF1^ (Tables 3 and 4). To enhance prediction accuracy, this study incorporated HR recovery during the step test as a predictive parameter. Including HR^0^ and exercise HR parameters increased the explained variance in VO₂max by 10.90% for Model^BMI3^ and 5.35% for Model^PBF3^, with SEE reductions of 7.06% and 4.51%, respectively. Compared to the most economical Model^BMI1^, Model^PBF3^ increased the explained variance in VO₂max by 17.38%, with a SEE reduction of 12.61%. These results indicate that incorporating exercise HR parameters significantly improves the accuracy of VO₂max prediction models based on biometric data. Moreover, PBF-based models consistently demonstrated higher accuracy than BMI-based models. Specifically, compared to Model^BMI1^, Model^PBF1^ increased the explained variance in VO₂max by 11.41% and reduced SEE by 8.48%; compared to Model^BMI2^, Model^PBF2^ increased the explained variance by 8.20% and reduced SEE by 7.00%; and compared to Model^BMI3^, Model^PBF3^ increased the explained variance by 5.84% and reduced SEE by 5.98% (Figure 1B). These findings align with previous studies indicating that PBF is a superior predictor of VO₂max compared to BMI 35,36,48. Therefore, when economic conditions permit, the public should consider using PBF-based models to estimate their VO₂max for greater accuracy. For individuals with limited resources, BMI-based models offer a more economical and practical alternative.

This study evaluated the agreement between the model and measured VO₂max using Bland-Altman analysis. The results showed that the PBF model had a bias of -0.35 ml·kg⁻¹·min⁻¹, indicating no significant systematic error. Additionally, the limits of agreement ranged from -9.53 to 8.82 ml·kg⁻¹·min⁻¹, demonstrating that most prediction errors fell within this range, reflecting greater stability compared to the BMI model. These findings support the PBF model as a more accurate VO₂max prediction tool, suitable for general health assessments and sports medicine applications. To validate the VO₂max prediction formulas developed in this study, cross-validation was performed. PRESS cross-validation analysis revealed minimal differences in R² (0.008-0.059) and SEE (0.023-0.280 ml·kg⁻¹·min⁻¹) between the training and testing groups across the six prediction models (Tables 3 & 4). These results confirm the validity of the VO₂max prediction models developed in this study. To further assess the validity and reliability of VO₂max prediction models categorized by BMI, Pearson correlation coefficients and ICCs were used. The results indicated that the predictive validity of Model^BMI1^, Model^BMI2^, and Model^BMI3^ was 0.772, 0.788, and 0.797, respectively, with corresponding reliability values of 0.743, 0.765, and 0.776 (Figure 2A). In contrast, for the PBF-based models, Model^PBF1^, Model^PBF2^, and Model^PBF3^ demonstrated predictive validity of 0.808, 0.818, and 0.823, and reliability values of 0.789, 0.801, and 0.807, respectively (Figure 2B). Compared to the BMI-based models, the PBF-based models showed improvements in predictive validity of 4.66%, 3.81%, and 3.26% and in reliability of 6.19%, 4.71%, and 4.00% for Model^PBF1^, Model^PBF2^, and Model^PBF3^, respectively (Figure 2C). In summary, the VO₂max prediction formulas developed in this study are feasible and reasonable based on the experimental results. However, this research has certain limitations and contributions. Specifically, the models were developed based on data from healthy adults aged 20-64 years, and thus their applicability to adolescents, elderly individuals, or patients with chronic conditions remains uncertain and should be interpreted with caution. Regarding limitations, the prediction formulas were developed based on healthy Taiwanese adults aged 20-64 years. Therefore, their applicability may be restricted for children, adolescents, older adults, or individuals with lower-limb impairments or limited mobility. On the other hand, the contributions of this study are noteworthy. The step test provides a simple, effective, space-efficient, and accessible method for assessing CRF in adults. Following cross-validation and tests of validity and reliability, the six prediction models were validated and can be utilized by the public to select a model suited to their individual circumstances, removing barriers to CRF assessment. The step test-based VO₂max prediction model proposed in this study provides a cost-effective and convenient method for cardiorespiratory fitness assessment, applicable to various clinical settings, including primary healthcare, cardiac rehabilitation, and chronic disease monitoring. This model can serve as an initial screening tool for cardiorespiratory fitness, particularly for populations with hypertension, diabetes, and chronic obstructive pulmonary disease (COPD), and can be utilized for personalized health monitoring through telemedicine technologies. Furthermore, compared to the 6-minute walk test (6MWT) and cardiopulmonary exercise testing (CPET), this model offers a shorter testing duration and simpler implementation, making it well-suited for resource-limited healthcare environments. Additionally, these formulas are not only practical for the Taiwanese population but also offer a viable CRF assessment option for individuals and researchers worldwide. The models established in this study can be adapted to various testing protocols, such as different step heights or frequencies, making them valuable for further research and development. Compared with other predictive methods for VO₂max estimation, such as the 3-minute progressive knee-ups and step test proposed by Chung et al 35, our method demonstrates several advantages. The current step test protocol requires less physical effort and is easier to implement without equipment for intensity progression. In contrast, protocols involving progressive movement or graded load may yield better predictive accuracy but require stricter standardization and more coaching. However, the performance-predictor relationships in such tests may be influenced by factors unrelated to CRF, such as coordination or muscular endurance. Our model, emphasizing heart rate recovery, offers a safe and efficient solution particularly suitable for large-scale screening or populations with limited mobility. Nevertheless, future studies may consider hybrid approaches to further enhance VO₂max prediction accuracy. This approach has the potential to improve global accessibility to CRF evaluation, enabling people worldwide to better understand their CRF and achieve effective data translation.

## Figures and Tables

**Table 1:** Physical characteristics of the participants. Values are presented as mean ± standard deviation (SD). BMI, body mass index; PBF, percent body fat; RHR, resting heart rate. * *p*-value was calculated from one-way ANOVA test between training group and testing group.

	Training group			Testing group			P*
	Female (*n* = 72)	Male (*n* = 68)	Total (*n* = 140)	Female (*n* = 28)	Male (*n* = 32)	Total (*n* = 60)	
Age (years)	41.49 ± 12.66	38.91 ± 10.29	40.24 ± 11.60	39.46 ± 12.52	39.41 ± 12.42	39.43 ± 12.36	0.661
Height (cm)	159.97 ± 5.54	173.27 ± 6.19	166.43 ± 8.87	159.50 ± 4.76	174.20 ± 6.64	167.34 ± 9.39	0.513
Body weight (kg)	58.08 ± 9.57	74.45 ± 11.16	66.03 ± 13.20	57.71 ± 8.93	76.02 ± 11.52	67.48 ± 13.82	0.486
BMI (kg/m^2^)	22.63 ± 2.96	24.76 ± 3.21	23.66 ± 3.25	22.70 ± 3.53	25.00 ± 3.10	23.93 ± 3.48	0.608
PBF (%)	30.00 ± 6.62	21.17 ± 6.13	25.71 ± 7.75	29.53 ± 7.41	21.68 ± 6.36	25.34 ± 7.87	0.759
VO_2_max (ml·kg^-1^·min^-1^)	27.09 ± 5.51	37.04 ± 7.62	31.92 ± 8.27	26.06 ± 6.21	36.09 ± 6.85	31.41 ± 8.23	0.689
RHR (bpm)	76.67 ± 10.32	75.01 ± 10.40	75.86 ± 10.36	77.07 ± 10.87	73.38 ± 12.49	75.10 ± 11.81	0.647

**Table 2:** Pearson correlations between measured VO2max and independent variables for the training group in the step test. (n = 140)

	VO_2_max	Age	Sex	BMI	PBF	RHR	HR^0^	HR^1^	HR^2^	HR^3^	HR^4^
Age	-0.409^**^	1									
Sex	0.603^**^	-0.111	1								
BMI	-0.104	-0.045	0.328^**^	1							
PBF	-0.738^**^	0.242^**^	-0.571^**^	0.397^**^	1						
RHR	-0.241^**^	0.026	-0.080	-0.033	0.141	1					
HR^0^	-0.063	0.163	0.095	0.155	0.044	0.072	1				
HR^1^	-0.110	0.289^**^	0.093	0.089	0.062	0.106	0.599^**^	1			
HR^2^	-0.188^*^	0.377^**^	0.102	0.078	0.055	0.057	0.539^**^	0.898^**^	1		
HR^3^	-0.245^**^	0.406^**^	0.050	0.068	0.094	0.059	0.514^**^	0.840^**^	0.947^**^	1	
HR^4^	-0.175^*^	0.281^**^	0.101	0.120	0.031	-0.009	0.438^**^	0.516^**^	0.697^**^	0.787^**^	1
HR^3-4^	-0.090	0.166	-0.083	-0.086	0.092	0.102	0.080	0.444^**^	0.319^**^	0.257^**^	-0.394^**^

Notes*.* BMI, body mass index; PBF, percent body fat; RHR, resting heart rate; HR^0^, HR at the beginning of exercise; HR^1^, HR at the 1st minute of exercise; HR^2^, HR at the 2nd minute of exercise; HR^3^, HR at the 3rd minute of exercise; HR^4^, HR at the 4th minute of post-exercise; HR^3-4^, difference between heart rate at the third minute during the exercise and the first minute post exercise during the Step test; *p < 0.05; ** *p* < 0.01.

**Table 3:** Multiple regression model (BMI-based) to predict VO_2_max (ml·kg^-1^·min^-1^).

VO_2_max (ml·kg^-1^·min^-1^)	B	Standard Error	*β*	*p* value
Model^BMI1^				
Constant	57.054	3.802		< 0.001
BMI (kg·m^-2^)	-0.868	0.148	-0.341	< 0.001
Age (years)	-0.249	0.040	-0.349	< 0.001
Sex (men = 1, women = 0)	11.150	0.967	0.676	< 0.001
R²	0.587			
Adjusted R^2^	0.577			
SEE (ml·kg^-1^·min^-1^)	5.3750			
SEE%	16.221			
R²*p*	0.611			
SEE*p*	5.0949			
Model^BMI2^				
Constant	68.668	4.879		< 0.001
BMI (kg·m^-2^)	-0.871	0.142	-0.343	< 0.001
Age (years)	-0.247	0.038	-0.346	< 0.001
Sex (men = 1, women = 0)	10.912	0.930	0.662	< 0.001
RHR	-0.152	0.042	-0.190	< 0.001
R²	0.622			
Adjusted R^2^	0.611			
SEE (ml·kg^-1^·min^-1^)	5.1554			
SEE%	16.156			
R²*p*	0.614			
SEE*p*	5.0746			
Model^BMI3^				
Constant	75.247	5.574		< 0.001
BMI (kg·m^-2^)	-0.860	0.138	-0.338	< 0.001
Age (years)	-0.200	0.041	-0.281	< 0.001
Sex (men = 1, women = 0)	10.910	0.907	0.662	< 0.001
RHR	-0.158	0.041	-0.197	< 0.001
Step test HR^1^	0.121	0.050	0.232	0.017
Step test HR^3^	-0.157	0.048	-0.324	0.001
R²	0.651			
Adjusted R^2^	0.635			
SEE (ml·kg^-1^·min^-1^)	4.9958			
SEE%	16.610			
R²*p*	0.592			
SEE*p*	5.2172			

Notes. B, unstandardized regression weights; β, standardized regression weights; BMI, body mass index; RHR, resting heart rate; HR^1^, HR at the 1st minute of exercise; HR^3^, HR at the 3rd minute of exercise; SEE, standard error of estimate; SEE%, SEE/mean of measured VO_2_max × 100; R^2^*p*, PRESS squared multiple correlation coefficient; SEE*p*, PRESS standard error of estimate.

**Table 4:** Multiple regression model (PBF-based) to predict VO_2_max (ml·kg^-1^·min^-1^).

VO_2_max (ml·kg^-1^·min^-1^)	B	Standard Error	*β*	*p* value
Model^PBF1^				
Constant	51.111	2.326		< 0.001
PBF (%)	-0.551	0.067	-0.517	< 0.001
Age (years)	-0.181	0.037	-0.253	< 0.001
Sex (men = 1, women = 0)	4.614	1.014	0.280	< 0.001
R²	0.654			
Adjusted R^2^	0.646			
SEE (ml·kg^-1^·min^-1^)	4.9190			
SEE%	15.495			
R²*p*	0.645			
SEE*p*	4.8668			
Model^PBF2^				
Constant	59.178	3.622		< 0.001
PBF (%)	-0.529	0.066	-0.496	< 0.001
Age (years)	-0.181	0.036	-0.255	< 0.001
Sex (men = 1, women = 0)	4.617	0.988	0.280	< 0.001
RHR	-0.113	0.040	-0.142	0.005
R²	0.673			
Adjusted R^2^	0.664			
SEE (ml·kg^-1^·min^-1^)	4.7945			
SEE%	15.336			
R²*p*	0.652			
SEE*p*	4.8171			
Model^PBF3^				
Constant	65.059	4.218		< 0.001
PBF (%)	-0.521	0.065	-0.489	< 0.001
Age (years)	-0.155	0.037	-0.217	< 0.001
Sex (men = 1, women = 0)	4.972	0.978	0.302	< 0.001
RHR	-0.114	0.039	-0.143	0.004
Step test HR^4^	-0.060	0.023	-0.131	0.011
R²	0.689			
Adjusted R^2^	0.677			
SEE (ml·kg^-1^·min^-1^)	4.6971			
SEE%	15.533			
R²*p*	0.643			
SEE*p*	4.8788			

Notes. B, unstandardized regression weights; β, standardized regression weights; PBF, percent body fat; RHR, resting heart rate; HR^4^, HR at the 4th minute of post-exercise; SEE, standard error of estimate; SEE%, SEE/mean of measured VO_2_max × 100; R^2^*p*, PRESS squared multiple correlation coefficient; SEE*p*, PRESS standard error of estimate.
